# The anxiety response of patients with severe psychiatric disorders to the recent public health crisis

**DOI:** 10.1186/s12888-024-05742-y

**Published:** 2024-04-23

**Authors:** Mohammadrasoul Khalkhali, Parsa Zarvandi, Mehrshad Mohammadpour, Seyed Mohsen Kheirkhah Alavi, Parnian Khalkhali, Hassan Farrahi

**Affiliations:** Kavosh Cognitive Behavior Sciences and Addiction Research Center, Department of Psychiatry, School of Medicine, Guilan University of Medical Sciences, Rasht, Iran

**Keywords:** Anxiety, Severe psychiatric disorders, COVID-19, Public health crisis

## Abstract

**Background:**

The devastating health, economic, and social consequences of COVID-19 may harm the already vulnerable groups, particularly people with severe psychiatric disorders (SPDs). The present study was conducted to investigate the anxiety response of patients with SPDs during the COVID-19 pandemic.

**Methods:**

A total of 351 patients with SPDs [Schizophrenia Spectrum (SSD), Bipolar (BD), Major Depressive (MDD), and Obsessive-Compulsive (OCD) Disorders] and healthy controls in Guilan province, Iran, throughout 2021–2022 were included in this cross-sectional analytical study. The anxiety response consisted of four concepts: COVID-19-related anxiety, general health anxiety, anxiety sensitivity, and safety behaviors. We conducted an unstructured interview and provided sociodemographic and clinical information. Also, the participants were asked to complete four self-report measures of the Corona Disease Anxiety Scale, the Anxiety Sensitivity Index–Revised, the Short Health Anxiety Inventory, and the Checklist of Safety Behaviors.

**Results:**

Analysis of variance showed a significant difference between the groups of patients with SPDs and the control group in COVID-19-related anxiety (F = 6.92, *p* = 0.0001), health anxiety (F = 6.21, *p* = 0.0001), and safety behaviors (F = 2.52, *p* = 0.41). No significant difference was observed between them in anxiety sensitivity (F = 1.77, *p* = 0.134). The Games-Howell test showed that the control group obtained a higher mean than the groups of people with BD (*p* < 0.0001), SSD (*p* = 0.033), and OCD (*p* = 0.003) disorders in COVID-19-related anxiety. The patients with MDD (*p* = 0.014) and OCD (*p* = 0.01) had a higher mean score than the control group in health anxiety. Tukey’s test showed that the mean of safety behaviors of the control group was significantly higher than the OCD group (*p* = 0.21). No significant difference was found between the groups of patients with MDD, BD, SSD, and OCD in terms of COVID-19-related anxiety, health anxiety, and safety behaviors.

**Conclusion:**

Anxiety response to health crisis is different in groups with SPDs and control group. The findings of this study suggest that although health anxiety is present in many of these patients during the pandemic, their anxiety response to the health crisis may be less than expected. There can be various explanations, such as pre-existing symptoms, low health literacy, and possible co-occurring cognitive impairment. The results of this study have many practical and policy implications in meeting the treatment needs of this group of patients during public health crises and indicate that their needs may not be compatible with the expectations and estimates that health professionals and policymakers already have.

## Background

The beginning of COVID-19 disease at the end of December 2019 in Wuhan, China, soon became a global pandemic and created numerous and vast crises in various areas of human life, such as the health, economic, and social [1]. Health crises disproportionately affect poor and vulnerable populations, including people with mental disorders [2]. COVID-19 pandemic did not affect the whole society equally, and there was a profound inequality in their adverse consequences for human life. People with mental disorders are prone to more harm and adverse outcomes [3]. A possible reason is that screening for medical comorbidities is less common in people with mental disorders, and they have higher mortality and a poorer prognosis than ordinary people when diagnosed with a disease [4]. Public health crises can cause recurrence or exacerbation of existing psychiatric disorders and, or physical diseases if people have a strong stress response to those crises compared to the general population [5]. Variation in response to crises, particularly global health crises, is also observed among people with psychiatric disorders [6]. Many studies sought to answer the question of how does COVID-19 pandemic affects different psychiatric disorders. Although a large part of the literature examines the impact of COVID-19 on the illness and well-being of people with non-SPDs [7, 8], few studies address the vulnerability of people with SPDs to COVID-19. At the same time, this group often deals with more harmful and adverse consequences in the face of environmental threats [2, 3].

People with SPDs typically experience many disparities in physical health [9]. Individuals with SPDs have a life expectancy of about 15 years less than the general population. The main reason is that people with SPDs are at high risk of physical illness [10]. Previous studies have focused on cardiovascular and metabolic diseases in patients with SPDs. However, due to the spread of epidemics and pandemics in the past years, infectious diseases have also been addressed as one of the vulnerabilities in these patients [9]. Six large-scale studies from Sweden (*N* = 195,565), Denmark (*N* = 144,321), Israel (*N* = 125,273), the UK (*N* = 447,296), England (*N* = 34,446), and the US (*N* = 7,348) showed an increased risk of the COVID-19 infection, hospitalization, and mortality for patients with SPDs, with no increased risk for other psychiatric disorders such as anxiety disorders [3, 9, 11–14]. A South Korean cohort study found that patients with SPDs had a slightly higher risk for severe clinical outcomes of COVID-19 than patients without psychiatric disorders [15]. The patients with schizophrenia spectrum disorder had a higher risk of mortality related to COVID-19. It is unclear to what extent patients with BD and MDD are at increased risk of death from COVID-19 [16]. Pandemics are often perceived as a life-threatening danger and correspondingly evoke emotional (anxiety, distress, helplessness), physical (physiological reactions), and behavioral (avoidance, safety behaviors, reassurance) responses to protect the person exposed to it [17]. Feeling anxious about health, having increased physical arousal, and performing behaviors to eliminate or reduce the relevant danger are among the expected responses in such times, ranging on a continuum from adaptive to non-adaptive health anxiety [18]. Unexpectedly, engaging in safety behaviors may not always have the effect of reducing health anxiety. Performing safety behaviors can have the opposite effect even in healthy people. In one experimental study [19], non-anxious subjects who were assigned to perform more safety behaviors reported more health anxiety compared to non-anxious subjects. After the outbreak of COVID-19, there were several reports of increased health anxiety (and one of its components, death anxiety) in the general population [20–22] and people with mental disorders [23].

Some findings led to the assumption that it is better to consider distinctions between general health anxiety and anxiety related to COVID-19 [24, 25]. In a time-course analysis of 12 single cases with the diagnosis of pathological health anxiety, Sauer and colleagues [24] found that COVID-19-related anxiety was significantly lower than anxiety related to other severe diseases (e.g., cancer). However, COVID-19-related anxiety was not significantly associated with anxiety related to other severe diseases or pre-COVID-19 health anxiety. Norbye and colleagues [25] in the study of 1012 participants with one or more measurements of health anxiety between 2015 and 2020 and, or during the COVID-19 pandemic (from 2020 to 2022), found no significant changes in health anxiety scores during the pandemic compared to the first two years of the pandemic in Norwegian adults. This finding means that general health anxiety has remained stable over time, although anxiety specific to COVID-19 was significantly higher. On the other hand, several researchers have emphasized that the threat of COVID-19 differs significantly from the threats caused by other infectious diseases such as influenza. As a result, we can expect more exaggerated reactions to public health crises like COVID-19 [26]. For reasons like these, it has been argued that anxiety caused by COVID-19 is different from classic health anxiety in some ways, and due to the high severity of symptoms and high contagiousness and mortality, the fear and caution of people may seem justified and cannot be considered simply pathological [27]. Others have considered it necessary to use other terms, such as corona phobia, to name and describe anxiety related to COVID-19 [28].

In addition, research indicates that some general vulnerability and trans-diagnostic factors play a role in the level of anxiety, including health anxiety, in all types of psychiatric disorders and ordinary people [29, 30]. One well-studied factor is anxiety sensitivity [23]. Anxiety sensitivity is a multidimensional construct that consists of fears of physical, cognitive, and social aspects of anxiety. Individuals with higher anxiety sensitivity believe their symptoms and physical arousal can have harmful and dangerous consequences [29]. Anxiety sensitivity correlates with anxious reactions to life-threatening events, particularly pandemics [30]. This factor affects anxiety response to previous pandemic threats such as H1N1 and Ebola, although some findings have not shown such an association [23].

Although it is yet unknown what factors exactly predispose individuals to exaggerated anxiety in the face of a public health crisis [26], most findings indicate that anxiety responses are significantly affected in both healthy populations and psychiatric patients and cause severe distress and suffering [31–33]. This critical situation has a significant effect on the symptomatology of psychiatric patients and functional impairment. Such health crises can be the catalyst for the onset of SPDs or exacerbate symptoms [34]. On the other hand, some symptoms of SPDs can make patients less aware of the risks of contracting diseases such as COVID-19 or increase their anxiety. In contrast, other symptoms like disorganization can seriously interfere with patients’ compliance with health instructions or restrictions [35]. According to the Mental Health Commission of Canada, the clinical and support needs of patients with SPDs were subordinated to public health priorities during the COVID-19 pandemic, and there were marked interruptions in providing services to them [36]. According to the World Health Organization survey, these problems have been happening on a larger scale in developing countries [37]. In addition, factors such as ethnic/racial issues, socio-economic status, and the pre-existing health status of these patients have increased the layers of inequality [37]. Extensive and relevant research is emphasized to better understand the psychological and behavioral responses of patients with SPDs [36]. During the recent pandemic, many burdens were imposed on health service infrastructure in many countries. The findings of such studies can be used in the development and implementation of mental health promotion strategies and prevention or intensive therapeutic efforts [38].

While people with SPDs appear more vulnerable to COVID-19 disease and its consequences, research on them is more limited [14, 39]. The previous studies have not sufficiently addressed the possible distinction between general health anxiety and anxiety related to COVID-19 and the differences between diagnostic groups with SPDs in psychological responses to the recent public health crisis. To our knowledge, this is the first study to distinguish between general health anxiety and COVID-19-related anxiety responses. We examined anxiety sensitivity as an anxiety-specific vulnerability factor and safety behaviors as one of the most common behavioral reactions in the face of a pandemic in people with SPDs. We compared variables as mentioned earlier among the four diagnostic groups and between patients with SPDs and control subjects. We hypothesized that the four diagnostic groups with SPDs would obtain higher scores than controls on self-report assessments of general health anxiety, COVID-19-related anxiety, anxiety sensitivity, and safety behaviors. We also hypothesized that the four diagnostic groups do not have significant differences in those variables.

## Methods

### Study design

This cross-sectional analytical study was conducted on 351 patients with four severe psychiatric disorders and healthy individuals in Guilan province, Iran, throughout 2021–2022. This study received ethical committee approval (Code: IR.GUMS.REC.1400.334) and all methods were performed in accordance with the Declaration of Helsinki.

### Participants

This study was conducted on patients aged 18 to 65 with severe psychiatric disorders (schizophrenia spectrum, bipolar, major depressive, and, obsessive-compulsive disorders) who had been referred to the outpatient clinics in Rasht in Guilan province (the second center of the outbreak of COVID-19 in Iran). The research sample was selected using convenience sampling and divided into four distinctive experimental groups and a control group: 43 patients with schizophrenia spectrum disorders (schizophrenia, schizoaffective, and schizophreniform), 51 patients with bipolar disorders (type 1 and type 2), 46 patients with major depressive disorder, 60 patients with obsessive-compulsive disorder and 151 participants without any psychiatric disorders. The participants of the control group were selected from the normal relatives of the patients or from among the people who declared their readiness to participate in the research project after seeing the advertisement in the local online social networks.

### Procedures

Participants were recruited from patients referred to the outpatient clinic of Shafa Hospital and one private psychiatry clinic. The inclusion criteria were passing at least six months since the final diagnosis of the disorder, undergoing treatment for psychiatric disorders, having at least ninth-grade education, and the absence of any psychiatric disorders in the control group. Exclusion criteria were being in the intoxication phase of substance use or the acute phase of the disorder. A clinical interview using the diagnostic criteria based on the fifth edition of the Diagnostic and Statistical Manual of Mental Disorders [40] was conducted by a faculty psychiatrist (the first author of the study) on all participants (patients and control participants). Then, two trained medical students administered the study tools regarding COVID-19 disease, general health anxiety, anxiety sensitivity, and safety behaviors on the participants in random order and following the safety precautions recommended by the World Health Organization. After implementing the study measures on 366 participants, we subjected the gathered information to a preliminary review (data screening) to check for accuracy and identify the outliers and any missing data. There were no missing data in the scores of all the measures. We identified the outliers through the standard z score, so 16 participants with ± 3 z score at least at one measure were excluded. Finally, the statistical analysis was performed on the remaining 351 participants. The participants were included in the study based on their primary diagnosis. Necessary points were provided to the participants and written consent was obtained from them.

### Measures

1. The Information Checklist:

This checklist contains information related to the clinical status of the patients, including current psychiatric diagnosis and COVID-19 disease, as well as information related to demographic variables such as age, gender, marital status, education, employment status, substance/drug use, history of COVID-19 infection in oneself or their relatives (first or second-degree).

2. Corona Disease Anxiety Scale (CDAS):

This scale was developed and validated in Iran in 2019 by Alipour et al. to measure corona-disease-related anxiety [41]. This scale consists of 18 items and two components (factors). Items 1 to 9 (such as “Thinking about corona disease makes me anxious” and “I am worried about the spread of corona disease to people around me”) measure psychological symptoms, and items 10 to 18 (“Thinking about Corona has disturbed my sleep” and “When I think about Corona, my body trembles”) measure physical symptoms of anxiety. Item is scored on a 4-point Likert scale (never = 0, sometimes = 1, most of the time = 2, and always = 3. Therefore, the score that individuals get on this scale will be between 0 and 54, where higher scores indicate a higher level of anxiety. In Alipour and colleagues’ study [41], the reliability of CDAS using Cronbach’s alpha was obtained for the first and second factor, and the total scale of 0.88, 0.86, and 0.92, respectively. Also, the correlation of the CDAS with the total score of the 28-item General Health Questionnaire (GHQ-28) and the subscales of anxiety, physical symptoms, impairment in social functioning, and depression was found to be 0.48, 0.51, 0.42, 0.33, and 0.27, respectively (*P* < 0.01).

3. Anxiety Sensitivity Index - Revised (ASI-R):

The ASI-R is a 36-item self-report scale that measures the fear of anxiety-related sensations based on beliefs about their harmful consequences, and has a four-factor structure: (1) fear of respiratory symptoms (items such as “You feel like you’re suffocating” and “You feel like you’re choking”), (2) fear of gastrointestinal symptoms (items such as “Your stomach is making loud noises” and “You feel bloated”), (3) fear of cardiac symptoms (items such as “Your heart pounds in your ears” and “Your heart is beating so loud that you can hear it”), and (4) fear of cognitive dyscontrol (items such as “You can’t keep your mind on a task” and “You have trouble remembering things”) [42]. Respondents indicate their level of agreement with each item on a Likert scale that ranges from meager (score 0) to very high (score 4). Therefore, the range of scores is between 0 and 144, and higher scores indicate greater anxiety sensitivity. Taylor and Cox reported the internal consistency coefficient for factors 1 to 4, 0.91, 0.86, 0.88, and 0.89, respectively. The correlation coefficient between the ASI-R and the anxiety sensitivity index was 0.94. Also, they reported the correlation of factors with each other in the range of 0.28 to 0.40 and with the total score of the ASI-R in the range of 0.66 to 0.77 [42]. In an Iranian study, the Cronbach’s alpha coefficient of the ASI-R was 0.91 [43].

4. Short Health Anxiety Inventory (SHAI):

This self-report inventory was developed and validated by Salkovskis et al. to assess worry about one’s health, awareness of bodily sensations or changes, and fear of disease consequences [44]. SHAI contains 18 items like “As a rule, I am not afraid that I have a severe illness,” “I do not have any difficulty taking my mind off thoughts about my health,” and “If I hear about an illness, I never think I have it myself.” Each item has four choices and the respondents must choose one of the sentences that best describes her/his. Scoring for each item is from 0 to 3, and a high score indicates higher health anxiety. Its retest reliability is 0.90. Cronbach’s alpha coefficient is reported from 0.70 to 0.82. Its test-retest reliability, internal consistency, and convergent validity were reported as 0.90, 0.70 to 0.82, and 0.72, respectively [44]. In an Iranian study, Cronbach’s alpha coefficient of 0.87 was reported for SHAI [45].

5. Checklist of Safety Behaviors:

This checklist contains 23 safety behaviors related to COVID-19 disease. The respondents are asked to mark each of the behaviors in this checklist if they do it. Examples of its items included: “I wash/disinfect my hands more often,” “I increasingly avoid public places/ events,” and “I increasingly avoid public transit (subway, tram, bus, train).” The behaviors included in this checklist were extracted from the checklists examined in two studies by Musche and colleagues [46] and Olatunji and colleagues [19].

### Data analysis

To analyze the data, after calculating the central and dispersion statistical indices, the skewness and kurtosis indices and the Shapiro-Wilk test were used to examine the normal distribution of the data. Levene’s test was used to examine the homogeneity of variances. Then, a one-way analysis of variance (ANOVA) was used to discover the difference between the study groups in the research variables. Finally, regarding the variables that were found to be different between study groups in ANOVA, Tukey HSD and Games-Howell post hoc tests were used. Tukey’s test was used to examine those variables whose homogeneity of variance was confirmed by Levine’s test, and Games Howell’s test was used for those that were not confirmed.

## Results

A total of 351 patients and healthy controls participated in our study. Demographic data classified by four subgroups of patients with severe psychiatric disorders and a control group are presented in Table 1. 27.9% of the participants had a history of COVID-19 infection; 7.1% had lived with people who were infected with COVID-19; and 59.8% of their relatives were infected with COVID-19.

**Table 1:** Description of demographic characteristics of patient and control groups

	MDD	BD	SSD	OCD	Control	Total
**N**	46	51	43	60	151	351
**Age [M (SD)]**	51.5 (14.11)	43.94 (13.7)	43 (11.25)	39.6 (11.69)	39.83 (12.58)	42.31 (13.19
**Gender [n (%)]**						
Male	12 (26.1)	16 (31.4)	23 (53.5)	21 (35)	37 (24.5)	109 (31.1)
Female	34 (73.9)	35 (68.6)	20 (46.5)	39 (65)	114 (75.5)	242 (68.9
**Marital Status [n (%)]**						
Single	5 (10.9)	15 (29.4)	18 (41.9)	24 (40)	60 (39.7)	122 (34.8)
Married	41 (89.1)	30 (70.6)	25 (58.1)	30 (60)	91 (60.3)	229 (65.2)
**Education [n (%)]**						
Under diploma degree	14 (30.4)	12 (23.5)	14 (32.6)	13 (21.7)	10 (6.6)	63 (17.9)
Diploma degree	20 (43.5)	17 (33.3)	8 (18.6)	22 (36.7)	29 (19.2)	96 (27.4)
Associates’ degree	3 (6.5)	4 (7.8)	7 (16.3)	4 (6.7)	10 (6.6)	28 (8)
Bachelor’s degree	9 (19.6)	16 (31.4)	10 (23.3)	15 (25)	61 (40.4)	111 (31.6)
Master’s degree	0 (0)	1 (2)	3 (7)	6 (10)	26 (17.2)	36 (10.3)
Doctoral degree	0 (0)	1 (2)	1 (2.3)	0 (0)	15 (9.9)	17 (4.8)
**Employment Status [n (%)]**						
Employed	6 (13)	17 (33.3)	19 (44.2)	19 (31.7)	64 (42.2)	125 (35.6)
Unemployed	29 (63)	33 (45.1)	21 (48.8)	37 (61.7)	36 (23.8)	146 (41.6)
Student	2 (4.3)	4 (7.8)	1 (2.3)	3 (5)	29 (19.2)	39 (11.1)
Retired	9 (19.6)	7 (13.7)	2 (4.7)	1 (1.7)	22 (14.6)	41 (11.7)
**Substance use [n (%)]**						
No	44 (95.7)	50 (98)	25 (58.1)	58 (96.7)	151 (100)	328 (93.4)
Yes	2 (4.3)	1 (2)	18 (41.9)	2 (3.3)	0 (0)	23 (6.6)
**Corona infection**						
Owns	8 (17.4)	7 (13.7)	12 (27.9)	12 (20)	59 (39.1)	98 (27.9)
In family	2 (4.3)	6 (11.8)	2 (4.7)	3 (5)	12 (7.9)	25 (7.1)
In relatives	23 (50)	25 (49)	27 (62.8)	34 (56.7)	101 (66.9)	210 (59.8)

Note: MDD: Major Depressive Disorder; BD; Bipolar Disorder; SSD: Schizophrenia Spectrum Disorder; OCD: Obsessive-Compulsive Disorder

Shapiro-Wilk test (*p* < 0.01) and kurtosis and skewness indices showed that the distribution of scores follows the normal distribution. Also, Levene’s test did not confirm the assumption of homogeneity of variances in COVID-19-related anxiety (*p* = 0.001) and health anxiety (*p* < 0.0001), however this assumption was confirmed in anxiety sensitivity (*p* = 0.056) and safety behaviors (*p* = 0.065) (Table 2). Therefore, the Games-Howell post hoc test was used to examine the differences in COVID-19-related anxiety and health anxiety, and Tukey’s test was used to examine the differences in anxiety sensitivity and safety behaviors.

**Table 2:** Normal distribution indices and homogeneity of variances test of scores in patient and control groups

	MDD (*n* = 46)	BD (*n* = 51)	SSD (*n* = 43)	OCD (*n* = 60)	Control (*n* = 151)
**Measures of variability [(Statistic (SE)]**					
Skewness	1.12 (0.350)	0.869 (0.333)	1.802 (0.361)	2.218 (0.309)	1.126 (0.197)
Kurtosis	0.547 (0.688)	0.526 (0.656)	3.456 (0.759)	6.932 (0.608)	1.921 (0.392)
**COVID-19-related anxiety [Sig.]**					
Shapiro-Wilk	0.001	0.004	0.001	0.001	0.001
Levene’s test			0.001		
**Health anxiety [Sig.]**					
Shapiro-Wilk	0.035	0.060	0.038	0.115	0.020
Levene’s test			0.001		
**Anxiety sensitivity [Sig.]**					
Shapiro-Wilk	0.112	0.099	0.241	0.299	0.055
Levene’s test			0.056		
**Safety behaviors [Sig.]**					
Shapiro-Wilk	0.099	0.006	0.015	0.001	0.020
Levene’s test			0.065		

Note: MDD: Major Depressive Disorder; BD; Bipolar Disorder; SSD: Schizophrenia Spectrum Disorder; OCD: Obsessive-Compulsive Disorder

In Table 3, the mean and standard deviation of COVID-19-related anxiety, health anxiety, anxiety sensitivity, and the number of safety behaviors of different groups of participants are presented. ANOVA showed that there is a significant difference between the groups of patients with psychiatric disorders and the control group in terms of COVID-19-related anxiety (F = 6.92, *p* = 0.0001), health anxiety (F = 6.21, *p* = 0.0001), and safety behaviors (F = 2.52, *p* = 0.41). However, no significant difference was observed between them regarding anxiety sensitivity (F = 1.77, *p* = 0.134).

**Table 3:** Mean and standard deviation of participants’ scores by patient and control groups and significance of difference between groups in scores (ANOVA)

	Groups [M (SD)]					F	Sig.	Partial η^2^
	MDD (*n* = 46)	BD (*n* = 51)	SSD (*n* = 43)	OCD (*n* = 60)	Control (*n* = 151)			
**COVID-19-related anxiety**	10.98 (8.97)	7.04 (5.62)	8.09 (9.58)	8.63 (7.11)	12.97 (9.31)	6.92	0.001	0.074
**Health anxiety**	21.04(11.62)	16.59(7.46)	19.32(11.70)	19.50 (9.30)	15.09 (6.51)	6.21	0.001	0.067
**Safety behaviors**	9.00 (6.14)	9.39 (6.70)	9.59 (6.40)	7.22 (5.14)	9.90 (5.39)	2.52	0.041	0.028
**Anxiety sensitivity**	57.09(31.67)	50.88(33.43)	49.84(29.56)	55.88(30.02)	46.79(24.98)	1.77	0.134	0.020

Note: MDD: Major Depressive Disorder; BD; Bipolar Disorder; SSD: Schizophrenia Spectrum Disorder; OCD: Obsessive-Compulsive Disorder

As shown in Table 4, the Games-Howell test showed a significant difference between the control group and the groups of people with BD (*p* < 0.001), SSD (*p* = 0.033), and OCD (*p* = 0.003) in terms of COVID-19-related anxiety, so that the mean score of the control group was higher. There was a significant difference between the control group and MDD (*p* = 0.014) and OCD (*p* = 0.01) groups in terms of health anxiety, so the mean score of control group was lower. Tukey’s test showed that the mean of safety behaviors of the control group was significantly higher than OCD group (*p* = 0.21). It should be noted that no difference was observed between the groups of MDD, BD, SSD, and OCD in terms of COVID-19-related anxiety, health anxiety, and safety behaviors.

**Table 4:** Significance of differences between the patient and control groups (Games-Howell & Tukey)

	MDD (*n* = 46)	BD (*n* = 51)	SSD (*n* = 43)	OCD (*n* = 60)	Control (*n* = 151)
**COVID-19-related anxiety**					
MDD	-	0.089	0.588	0.593	0.686
BD	0.089	-	0.969	0.681	0.001
SSD	0.588	0.969	-	0.998	0.033
OCD	0.593	0.681	0.998	-	0.003
Control	0.686	0.001	0.033	0.003	-
**Health Anxiety**					
MDD	-	0.184	0.957	0.947	0.014
BD	0.184	-	0.678	0.362	0.706
SSD	0.957	0.678	-	1.000	0.171
OCD	0.947	0.362	1.000	-	0.010
Control	0.014	0.706	0.171	0.010	-
**Safety Behaviors**					
MDD	-	0.997	0.937	0.516	0.887
BD	0.997	-	0.990	0.281	0.983
SSD	0.937	0.990	-	0.127	1.000
OCD	0.516	0.281	0.127	-	0.021
Control	0.887	0.983	1.000	0.021	-

Note: MDD: Major Depressive Disorder; BD; Bipolar Disorder; SSD: Schizophrenia Spectrum Disorder; OCD: Obsessive-Compulsive Disorder

## Discussion

The recent pandemic has had devastating health, economic, and social consequences in many societies. These consequences harmed vulnerable groups, particularly people with SPDs [3]. In the present study, we examined the anxiety response of four groups of patients with SPDs in the face of the recent global health crisis compared to each other and people without any psychiatric disorder. The results of this study showed that, except for anxiety sensitivity, there was a significant difference between patients with SPDs and the control group in COVID-19-related anxiety, health anxiety, and safety behaviors. Unexpectedly, the control group had significantly higher COVID-19-related anxiety than patients with BD, SSD, and OCD, while patients with MDD and OCD had significantly higher health anxiety than the control group. The number of safety behaviors of the control group was significantly higher than that of the OCD group. No difference was observed between the four diagnostic groups in COVID-19-related anxiety, health anxiety, and safety behaviors.

Many meta-analytic and systematic review studies have shown an increase in anxiety and depression in the general population in dealing with the recent pandemic [47–49]. It was expected that patients with SPDs, considering their higher sensitivity to stress, would show more anxiety response when faced with the numerous health, economic, and social challenges of COVID-19. The findings of this study showed the opposite. The patients with SPDs, except for MDD, reported less COVID-19-related anxiety than the control group, while regarding health anxiety, two groups of patients with SPDs (MDD and OCD) showed higher health anxiety. Although these findings are consistent with the findings of some studies [50], most previous studies have reported that psychiatric patients, especially those with SPDs, experienced more anxiety than the general population [31–33]. One explanation is that pre-existing symptoms exacerbated their anxiety in the face of the pandemic [51].

The COVID-19 pandemic has become a global threat to mental health due to its contagiousness and high mortality [2, 3]. The news coverage of the pandemic was worldwide, and many rumors were spread about it [23]. On the other hand, World Health Organization and healthcare policymakers have equally required everyone to follow health and safety recommendations [46]. All this caused the consequences of the pandemic to become universal, and its psychological reactions spread to many people without psychiatric disorders. Stressful factors such as fear of death, fear of losing loved ones, loss of social connection, and loss of employment may even cause severe psychological problems in formerly healthy people [52]. Some studies reported that healthy individuals had higher levels of anxiety and depression than individuals with SPDs during COVID-19 health crisis [51]. What further strengthens this interpretation is that participants without psychiatric disorders performed more safety behaviors in response to the pandemic than patients with OCD, or did not differ in this respect from patients with SSD, BD, and MDD. There was no significant difference in anxiety sensitivity, as a general trans-diagnostic factor of vulnerability to experience anxiety between them [23]. This overall increase in anxiety and depression responses to COVID-19, which causes worsening psychological reactions in the general population, can be an expression of the ceiling effect [51, 53]. Such an effect may cause the previously apparent difference between the two populations to diminish and possibly disappear during periods of the public health crisis.

There are various reasons for the lower manifestation of COVID-19-related anxiety and safety behaviors in patients with SPDs. Some have suggested that the lives of patients with psychiatric disorders are already troubled by many challenges, and the outbreak of a global health crisis has had more adverse effects on the lives of non-disordered individuals [50, 51]. In other words, the most significant anxiety, interference, and restrictions caused by the pandemic have been created for those who had a less troublesome life before [51]. For example, many people with mental disorders may have previously experienced isolation and lack of a suitable social network, as a result, the “social distancing” recommended by the healthcare system and the resulting psychological consequences do not seem to be central problem for them [50]. Patients with SPDs were struggling with their own problems and symptoms, and there was a mismatch between public concerns related to Covid-19 and their own. For example, the fear of being infected with a virus was not important to a patient with OCD because she was afraid of being infected with toxic substances. Alternatively, a patient with BD may be more preoccupied with having an affair or tension with a partner than with health concerns. Neuropsychological deficits and symptoms may also interfere with patients’ understanding of health risk factors and cause inappropriate responses to them [54].

Symptoms include an exaggerated sense of well-being and self-confidence (euphoria), amotivation, and disorganized thinking can cause such interferences in the lives of patients with BD, MDD, and SSD, respectively [40]. As a result, amotivation in a patient with MDD can lead to neglecting self-protection or visiting a doctor if necessary, and disorganized thinking or delusional thinking in a patient with SSD can make her refuse to use a mask [55]. Also, since many patients with SPDs may have underlying cognitive impairments in information processing and proper planning and action [54], it has been suggested that cognitive deficits, along with other factors, such as low education level, cause a decrease in their health literacy and directly interfere with proper *hygiene behaviors* [15, 50]. Therefore, as much as the above factors affect the emotional response of patients with SPDs to a public health crisis, it can be necessary for clinicians to make revisions in their strategies for managing health care, especially during public health crises [56]. In other words, they should develop and implement therapeutic and educational programs according to the real needs of patients and pay attention to critical needs that were not the subject of the present study.

In addition, our results regarding the higher level of health anxiety in two groups of patients with SPDs (MDD and OCD) compared to control group may confirm the view of clinicians who believe that as long as the anxiety response to the threat of COVID-19 pandemic is possibly justified, it is better to distinguish between the pandemic-related anxiety and general or classic health anxiety. During a global pandemic, it may be reasonable for people to attribute their anxiety symptoms to corona virus infection and show considerable reactions to it. In the end, this difference is of degree and not of nature [27]. A longitudinal study of the level of health anxiety from 2015 to 2022 in Norway based on several measurements suggests that health anxiety remains stable despite the recent global health crisis [25]. All this indicates that despite the increase in the anxiety response of the general population to COVID-19, the previous level of health anxiety in patients with disorders such as OCD and MDD continues to exist as before. This finding is inconsistent with the findings of other studies that report a positive association between health anxiety and anxiety specific to COVID-19 virus in the general population [57, 58]. According to the sum of the above findings and interpretations, some researchers have suggested that new methods are needed to identify demographic and clinical indicators of vulnerability to the onset and/or exacerbation of common psychiatric symptoms such as anxiety to help prevent distress caused by public health crises [51, 53].

### Limitations

Our study has several limitations. First, this study was carried out under epidemic conditions and quarantine rules. Health restrictions created difficulties for access to patients and research activities. Some patients did not want to participate in the research due to health concerns. We reduced some of these limitations by using video interviews. Second, our sample was small in any four patient groups, and, as a result, that may hinder both clinical significance and generalizability of the results. Third, due to the limitations caused by social distancing during the pandemic, we used four self-report measures to evaluate different aspects of the anxiety response. These measures are widely used, and many studies have confirmed their psychometric properties [41–45], although the findings obtained from clinical interviews by clinicians can provide more objective and accurate information. Finally, there are ethnic communities in Guilan province. Some studies have shown that there is a considerable ethnic disparity in contracting COVID-19 disease or worsening its symptoms. African Americans with MDD, BD, and schizophrenia had a higher risk of contracting COVID-19 than Caucasians after controlling for medical conditions, suggesting that social, behavioral, and lifestyle factors related to ethnicity may also play a significant role in health inequalities [55]. Despite the importance of the effect of ethnicity and race on the experience of psychiatric and medical diseases [59], to our knowledge, there is no valid and reliable research data on a national or regional scale that shows the pattern of psychiatric disorders, particularly SPDs, in terms of different ethnicities and races living in Iran. In other words, although there is data about the pattern of psychiatric disorders in different provinces of Iran, due to various reasons, these disorders are not accurately reported according to ethnicity and race. Therefore, we cannot determine the effect of ethnicity on the anxiety response of patients with SPDs. In this sense, it is necessary to pay more attention to this category in future studies.

### Implications for future practice and research

The findings of this study have important implications for the investigation of health-related anxiety in patients with SPDs. First, due to the considerable heterogeneity in psychiatric disorders and their psychological and behavioral manifestations, it is recommended to examine health anxiety by considering various subgroups of the SPDs in future studies. That is especially true of OCD. The few existing studies indicate that patients with OCD had heterogeneous responses to health problems caused by COVID-19 [51]. The washing-contamination subgroup is expected to experience a greater fear of infection by a virus [23]. Although a study showed that there was no difference between different OCD subgroups in terms of anxiety and depression symptoms, and no significant difference was observed between healthy controls and OCD patients [51], a narrative review of studies indicated that obsessive-compulsive symptoms, especially hand washing, increased during the pandemic [60]. Further studies in the future can provide valuable information about the anxiety response pattern of OCD patients and its subtypes in the face of a global health crisis. Second, managing health-related problems requires intact cognitive abilities and adequate health literacy [55]. Considering the findings related to the possible deficiency of these factors in patients with SPDs [55], it is critical to pay sufficient attention to such factors in further studies and care management policies. Third, the findings of previous studies indicate that patients with SPDs have more vulnerability and mortality in COVID-19 pandemic [2, 3]. The findings of the present study showed that the patients with SPDs performed less safety behaviors compared to healthy people that has important implications for managing health-related behaviors in this group. It is essential to develop training programs for those patients and improve their inability to comply with mandatory health behaviors.

## Conclusion

The public health crisis has global effects on various aspects of human life. Patients with severe psychiatric disorders may show a considerable anxiety response to such a crisis due to their high vulnerability to stress. The findings of this study showed that there is a significant difference between the patients with SPDs and healthy individuals in terms of many components of the anxiety response to the recent public health crisis, and contrary to our expectations, healthy individuals experience more anxiety related to Covid-19. Although health anxiety persists in many of these patients during the pandemic, their anxiety response to the health crisis may be less than expected due to various factors such as pre-existing symptoms, low health literacy, and possible co-occurring cognitive impairment. It is reasonable to consider this level of anxiety response by patients with SPDs compared to individuals without psychiatric disorders. These findings can have many implications for research and health policy. In particular, they show that clinicians should expect a different pattern of anxiety reactions when dealing with patients and healthy people and consider tailored educational and therapeutic interventions.

## Declarations

### Data reproducibility

The dataset presented in the study is available on request from the corresponding author during submission or after publication.

## Abbreviations

SPDs: Severe psychiatric disorders
SSD: Schizophrenia Spectrum disorder
MDD: Major depressive disorder
BD: Bipolar disorder
OCD: Obsessive-compulsive disoder
